# EMixed: Probabilistic Multi-Omics Cellular Deconvolution of Bulk Omics Data

**DOI:** 10.6339/25-jds1170

**Published:** 2025-02-26

**Authors:** Manqi Cai, Kangyi Zhao, Penghui Huang, Juan C. Celedón, Chris McKennan, Wei Chen, Jiebiao Wang

**Affiliations:** 1 Department of Biostatistics and Health Data Science, University of Pittsburgh, USA; 2 Department of Statistics, University of Pittsburgh, USA; 3 Department of Pediatrics, University of Pittsburgh Medical Center Children’s Hospital of Pittsburgh, USA

**Keywords:** cellular deconvolution, DNA methylation, EM algorithm, gene expression, latent Dirichlet allocation, multi-omics

## Abstract

Cellular deconvolution is a key approach to deciphering the complex cellular makeup of tissues by inferring the composition of cell types from bulk data. Traditionally, deconvolution methods have focused on a single molecular modality, relying either on RNA sequencing (RNA-seq) to capture gene expression or on DNA methylation (DNAm) to reveal epigenetic profiles. While these single-modality approaches have provided important insights, they often lack the depth needed to fully understand the intricacies of cellular compositions, especially in complex tissues. To address these limitations, we introduce EMixed, a versatile framework designed for both single-modality and multi-omics cellular deconvolution. EMixed models raw RNA counts and DNAm counts or frequencies via allocation models that assign RNA transcripts and DNAm reads to cell types, and uses an expectation-maximization (EM) algorithm to estimate parameters. Benchmarking results demonstrate that EMixed significantly outperforms existing methods across both single-modality and multi-modality applications, underscoring the broad utility of this approach in enhancing our understanding of cellular heterogeneity.

## 1 Introduction

Tissue-level quantification of omics has gained popularity in the last decades because of its mature technology and affordable cost. Numerous studies on tissue-level omics, such as gene expression and DNA methylation (DNAm), provide rich resources to help answer interesting biological questions. However, bulk omics data are generated from a mixture of myriad cells, and thus tissue-level analyses are confounded by cellular heterogeneity and cell-type-specific (CTS) signals are obscured. Laborious technologies such as flow cytometry and immunohistochemistry can help measure cell type compositions, but they are costly and remain challenging to count cells in solid tissues. As a cost-efficient computational alternative, cellular deconvolution has been studied to decipher the cell type composition of bulk omics data, enabling us to remove the cellular heterogeneity confounding factors and infer CTS signals from bulk tissue data (Jaffe and Irizarry, 2014; Zheng et al., 2017; Avila Cobos et al., 2020).

To our knowledge, nearly all existing reference-based deconvolution methods are designed for single omics data (Cai et al., 2022; Avila Cobos et al., 2020; Jeong et al., 2022) or deconvolve each omics data type separately (Chang et al., 2019). While single-omics deconvolution has been helpful, each omics data modality has its disadvantages and only quantifies partial information from biological samples. For example, RNA-seq provides dynamic insights into gene expression, but its data can be noisy and influenced by technical noise and transient fluctuations. On the other hand, DNA methylation (DNAm) offers epigenetic information that is more stable but lacks the short-term temporal resolution provided by RNA-seq. Moreover, specific CTS markers may appear weak in some omics data types due to technical variability. As a consequence, cellular deconvolution in solid tissues like the brain shows moderate performance using single omics in real data benchmarking (Patrick et al., 2020). Multi-omics deconvolution addresses these limitations by integrating complementary data types, leveraging the strengths of each modality. For example, combining RNA-seq and DNAm data can enhance the resolution of cellular heterogeneity, as each omic data type provides distinct yet complementary views of the biological system. Multi-omics approaches also improve the robustness of deconvolution results by mitigating biases or noise that may dominate when using a single modality. Therefore, there is a need to develop new methods to jointly leverage information across multi-omics data and improve cellular fraction estimates.

To address these challenges, we propose EMixed, a deconvolution framework designed to integrate information across multiple omics data types. EMixed is based on latent Dirichlet allocation (LDA), a probabilistic model traditionally used in text analysis but increasingly applied to biological data to uncover latent structures, such as cellular composition (Zhu et al., 2018; Swapna et al., 2023; Chu et al., 2022). Unlike traditional methods that focus on a single omics modality, EMixed models both RNA and DNAm data, utilizing the complementary aspects of each to produce more accurate estimates of cellular composition. By employing an expectation-maximization (EM) algorithm, EMixed integrates data from different modalities, addressing the limitations posed by variability inherent in individual omics datasets.

LDA-based deconvolution methods, like EMixed, provide a robust framework for modeling the underlying structure of complex biological datasets. These methods assume that the observed data are mixtures of hidden components, corresponding to different cell types in the context of tissue analysis. Unlike traditional LDA-based methods, which often use computationally intensive Markov chain Monte Carlo sampling to estimate parameters, EMixed introduces an innovative computational strategy that directly maps expected latent variable values to the maximum likelihood estimator (MLE) of relevant parameters. We show this dramatically improves computational efficiency without sacrificing statistical fidelity.

EMixed further extends its utility by integrating both RNA and DNAm results, enabling a multifaceted analysis that broadens the scope of cellular deconvolution. This integrated approach is particularly useful in complex tissues, such as the brain. By combining diverse data sources, EMixed improves the accuracy of cellular fraction estimates and provides deeper insights into tissue biology. Benchmarking results demonstrate that EMixed performs well across various datasets and conditions, underscoring its potential utility in both research and clinical settings. By integrating multiple layers of biological information and leveraging the strengths of LDA-based modeling, EMixed advances the field of cellular deconvolution, offering a more precise and comprehensive approach to tissue data analysis.

## 2 Methods

**Figure 1:**
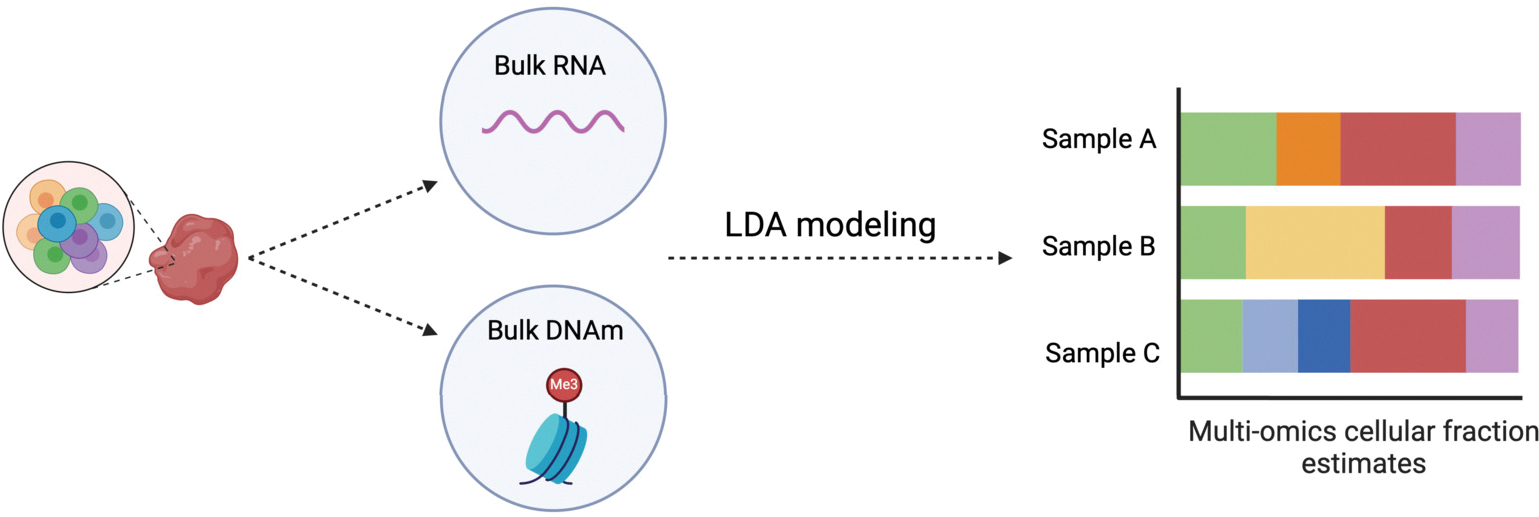
Overview of the proposed EMixed framework. Bulk RNA and bulk DNA methylation (DNAm) data are derived from the same biological tissue sample, consisting of mixed cell types. These two data modalities are modeled separately using latent Dirichlet allocation (LDA) to estimate cellular fractions. The multi-omics cellular fraction estimates, as illustrated on the right, represent the deconvolution results for different samples, with each color corresponding to a different cell type. EMixed integrates both RNA and DNAm data to provide a more accurate and comprehensive estimate of cellular composition.

The conceptual framework of EMixed is shown in Figure 1. EMixed is a multi-omics deconvolution method designed to analyze heterogeneous biological samples by leveraging both RNA-seq (bulk RNA) and DNA methylation (bulk DNAm) data derived from the same sample. Unlike traditional approaches that focus on a single modality, EMixed employs LDA modeling separately for each modality—RNA-seq and DNAm—thereby allowing for a more refined and accurate estimation of cellular composition. In Figure 1, we illustrate this process where bulk RNA-seq and DNAm data are collected from the same biological sample, which contains a mixture of cell types. The LDA models are applied individually to the RNA-seq and DNAm data, capturing distinct but complementary information from each modality. The results from both models are then integrated to estimate cellular fractions.

### 2.1 Deconvolving Bulk RNA-Seq Data

We draw inspiration from the LDA model for RNA-seq data introduced in Zhu et al. (2018). This model shares a close conceptual relationship with the LDA framework in topic modeling. Specifically, by drawing an analogy in which a gene read corresponds to a word, a cell type corresponds to a topic, and a bulk sample corresponds to a document, the parallels between the two approaches become evident. This model exhibits a conceptual parallel to the LDA framework in topic modeling. Accordingly, the model can be reformulated as a mixture of multinomials by introducing augmented latent variables $Z_{rn}$ (cell type allocation) and $d_{rn}$ (gene expression allocation) for RNA read r in bulk sample n. $Z_{rn}=\left[Z_{rn,1},\ldots ,Z_{rn,K}\right]$ is coupled with the constraint that $\sum_{k=1}^{K}\;Z_{rn,k}=1$, where $Z_{rn,k}$ represents an indicator that the $r^{th}$ RNA read from tissue sample n is originated from a type k cell. By definition, if $Z_{rn,k}=1$, $\delta_{rn}=k$, where k ranges from 1 to K. Similarly, $d_{rn}=\left[d_{rn,1},\ldots ,d_{rn,I}\right]$ is coupled with the constraint that $\sum_{i=1}^{I}\;d_{rn,i}=1$, where $d_{rn,i}$ represents an indicator that the $r^{\mathrm{th}}$ RNA read from tissue sample n is originated from gene i.

For each sample n, we have:

(1) $$\begin{array}{ll} Z_{rn}\overset{\text{i.i.d.}}{∼}Multinomial\left(1,\theta_{n}\right), & r=1,\ldots ,R_{n},\\ d_{rn}\overset{\text{indep.}}{∼}Multinomial\left(1,A_{\cdot \delta_{rn}}\right), & r=1,\ldots ,R_{n},\\ X_{in}=\sum_{r=1}^{R_{n}}\;d_{rn,i}, & i=1,\ldots ,I,\;n=1,\ldots ,N, \end{array}$$

where $R_{n}$ is the number of total read counts in bulk sample n and $X_{\mathrm{in}}$ represents RNA-seq counts of gene i in bulk sample n. $\theta_{n}=\left[\theta_{n1},\cdots ,\theta_{nK}\right]$ is a K×1 vector of cell type compositions that are non-negative and sum to one for K cell types. A is the profile matrix with the dimension of I genes by K cell types, obtained by normalizing the average cell type-specific gene expression matrix based on sequencing depths. The column sum of A is one.

While the LDA model presupposes observations of $d_{rn}$, representing the actual words in a document, only the final counts $X_{\mathrm{in}}$ are observable in RNA-seq data. Additionally, the sequencing depths $R_{n}$ tend to be substantial in real-world data scenarios, rendering the management of $Z_{rn}$ and $d_{rn}$ exceedingly computationally intensive. To facilitate the computation, we aggregate reads to genes and further define that

$${\tilde{Z}}_{in,k}\;:=\sum_{r:d_{rn,i}=1}Z_{rn,k},\;{\tilde{Z}}_{in}\;:=\left({\tilde{Z}}_{in,k}\right)\in R^{K}.$$

Based on the bulk data likelihood, we can derive that:

$${\tilde{Z}}_{in}|\theta ,X∼\;\mathrm{Multinomial}\;\left(X_{in},\frac{A_{i\cdot }⊙\theta_{n}}{\sum_{k=1}^{K}\;A_{ik}\theta_{nk}}\right),$$

where ⊙ denotes the element-wise multiplication.

The E-step for RNA-seq can be represented as:

(2) $$E\left({\tilde{Z}}_{in,k}|\theta ,X\right)=\psi_{in,k}^{\left(t\right)}=\frac{X_{in}A_{ik}\theta_{nk}^{\left(t-1\right)}}{\sum_{k^{\prime }=1}^{K}\;A_{ik^{\prime }}\theta_{k^{\prime }n}^{\left(t-1\right)}}.$$

The M-step is:

(3) $${\overset{ˆ}{\theta }}_{nk}=\frac{\sum_{i=1}^{I}\;\psi_{in,k}^{\left(t\right)}}{\sum_{i=1}^{I}\;\sum_{k=1}^{K}\;\psi_{in,k}^{\left(t\right)}}.$$

For parameter estimation, we focus on the interpretation and application of the cellular fraction parameter θ. We model RNA transcripts directly using an LDA model. Thus, θ should be understood as the proportion of transcripts attributed to specific cell types within the tissue (i.e., RNA fractions). However, when estimating actual cell fractions, we must account for the differing transcript abundances across cell types, which is represented by a cell size vector $S\in R_{+}^{K}$. S can either be provided or estimated based on the average library sizes of the cell types. To adjust for these differences in transcript abundance, we update the cell fractions at each iteration by estimating them as:

$$\theta_{nk}^{\mathrm{cell}}=\frac{\theta_{nk}/S_{k}}{\sum_{k^{\prime }=1}^{K}\;\left(\theta_{nk^{\prime }}/S_{k^{\prime }}\right)},$$

where $\theta_{nk}^{\mathrm{cell}}$ represents the adjusted cell fractions that properly reflect the varying contributions of transcripts from different cell types.

### 2.2 Deconvolving Bulk DNA Methylation Data

Building upon Psioda (2016), which focused on sequencing-based DNAm read counts and sorted-cell references, we have refined and expanded EMixed’s derivation to incorporate single-cell DNA methylation (scDNAm) signatures and extend it to array-based bulk DNAm data. We address key limitations of the original framework in two aspects: 1) Extension to continuous array-based data: by adapting the methodology to handle array-based DNAm data, we make it compatible with widely used platforms, broadening its utility beyond sequencing datasets. 2) Integration of scDNAm signatures: incorporating single-cell DNAm signatures enhances the granularity and precision of deconvolution, allowing for improved resolution in characterizing cellular heterogeneity.

We first introduce Psioda (2016)’s deconvolution model that targets individual DNA molecules to determine methylation in bulk DNAm sequencing data. It models a sample-specific latent multinomial distribution, determining the cell type for each DNA molecule. Let k=1,…,K denote a cell type, g=1,…,G for a DNAm locus, n=1,…,N for a mixed tissue sample, and $d=1,\ldots ,N_{ng}$ for a DNA molecule. There are G DNAm loci for analysis, within which K cell types form each mixed tissue sample. Here, a locus refers to a single CpG dinucleotide.

The model for cell type allocation is expressed as a latent multinomial distribution.

(4) $$\begin{array}{cc} M_{ngd}\overset{\mathrm{i.i.d.}}{∼}Multinomial\left(1,\theta_{n}\right), & g=1,\ldots ,G,d=1,\ldots ,N_{ng},\\ y_{ngd}\overset{\mathrm{indep.}}{∼}Bernoulli\left(\pi_{g\delta_{ngd}}\right), & g=1,\ldots ,G,d=1,\ldots ,N_{ng}. \end{array}$$

Under our model, we assume that DNAm and RNA samples, representing two omics layers from the same biological source, share the same cell type compositions $\theta_{n}$. In this process, we introduce the latent indicator variables $M_{ngd}=\left[M_{ngd,1},\ldots ,M_{ngd,K}\right]$ coupled with the constraint that $\sum_{k=1}^{K}M_{ngd,k}=1$, where $M_{ngd,k}$ represents an indicator that the $d^{th}$ DNA molecule covering locus g from tissue sample n is originated from a type k cell. By definition, if $M_{ngd,k}=1,\;\delta_{ngd}=k$, where k ranges from 1 to K. The cell type allocation model is inherently derived from the premise that the origin of each DNA molecule is influenced by the proportional abundance of its corresponding cell type within a heterogeneous tissue sample. This model intuitively links the molecular origin to the prevalent cellular composition of the tissue.

In the Bernoulli methylation model, $y_{ngd}$ is an indicator that the DNA molecule is methylated and $\pi_{gk}$ is the known signature methylation probability for type k cells at locus g that can be easily derived from single-cell or sorted-cell DNAm references and $\pi_{g}=\left[\pi_{g1},\cdots ,\pi_{gK}\right]$.

To estimate the parameters, we derive an EM algorithm, the latent indicator variables $M_{ngd,k}$, conditioned on the methylation status $y_{ngd}=1$, the latent indicator variables $M_{ngd,k}$ are replaced with their expected values, and similarly, $M_{ngd,k}$ conditioned on $y_{ngd}=0$ are replaced with their expected values. At the $t^{\mathrm{th}}$ iteration, we can derive the expected value of the latent variable $M_{ngd,k}$ as

(5) $$E\left[M_{ngd,k}|y_{ngd}=1,\theta_{n}^{\left(t\right)},\pi_{g}^{\left(t\right)}\right]=\psi_{ngk,1}^{\left(t\right)}=\frac{\theta_{nk}^{\left(t\right)}\pi_{gk}^{\left(t\right)}}{\sum_{i=1}^{K}\;\theta_{ni}^{\left(t\right)}\pi_{gi}^{\left(t\right)}};$$

similarly, we can get

(6) $$E\left[M_{ngd,k}|y_{ngd}=0,\theta_{n}^{\left(t\right)},\pi_{g}^{\left(t\right)}\right]=\psi_{ngk,0}^{\left(t\right)}=\frac{\theta_{nk}^{\left(t\right)}\left(1\;-\;\pi_{gk}^{\left(t\right)}\right)}{\sum_{i=1}^{K}\;\theta_{ni}^{\left(t\right)}\left(1\;-\;\pi_{gi}^{\left(t\right)}\right)}.$$

Given $D_{ng}$, which denotes the total count of DNA molecules covering locus g in the heterogeneous tissue sample n, and considering that the parameter set $\theta_{n}$ is influenced solely by sample n and cell type k, it can be inferred that $\theta_{n}$ is based on $D_{ng}$ independent observations for the computation of $\theta_{nk}^{\left(t\right)}$. Returning to Equation 4, which addresses the singular version of the problem, this setup aligns with the task of identifying the MLE of multinomial distributions. The expression $\sum_{n=1}^{N}\sum_{d=1}^{D_{ng}}M_{ngd,k}log\;\theta_{nk}$ in the log-likelihood function, coupled with the constraint $\sum_{k=1}^{K}M_{ngd,k}=1$ and considering the proportion Lagrange multiplier, guides us toward the solution:

(7) $$\theta_{nk}^{\left(t+1\right)}=\frac{\sum_{g=1}^{G}\;\psi_{ngk,1}^{\left(t\right)}\sum_{d=1}^{D_{ng}}\;y_{ngd}+\sum_{g=1}^{G}\;\psi_{ngk,0}^{\left(t\right)}\left(D_{ng}-\sum_{d=1}^{D_{ng}}\;y_{ngd}\right)}{\sum_{k=1}^{K}\;\sum_{g=1}^{G}\;\psi_{ngk,1}^{\left(t\right)}\sum_{d=1}^{D_{ng}}\;y_{ngd}+\sum_{k=1}^{K}\;\sum_{g=1}^{G}\;\psi_{ngk,0}^{\left(t\right)}\left(D_{ng}-\sum_{d=1}^{D_{ng}}\;y_{ngd}\right)}.$$

For sequencing-based DNAm data, we observe binary methylated/unmethylated status $y_{ngd}$ for $D_{ng}$ total covered counts. Instead, for array-based bulk DNAm data, we only observe the methylation probability, which is equivalent to $\beta_{ng}=\sum_{d=1}^{D_{ng}}y_{ngd}/D_{ng}$ in sequencing data. To extend the EM algorithm to array bulk data, it is reasonable to assume that $D_{ng}$ is a large constant that remains the same for all loci g and $D_{ng}=D_{n}$. With this, we can derive that

(8) $$\begin{array}{l} \theta_{nk}^{\left(t+1\right)}\;=\frac{\sum_{g=1}^{G}\;\psi_{ngk,1}^{\left(t\right)}\sum_{d=1}^{D_{ng}}\;y_{ngd}/D_{n}+\sum_{g=1}^{G}\;\psi_{ngk,0}^{\left(t\right)}\left(1\;-\;\sum_{d=1}^{D_{ng}}\;y_{ngd}/D_{n}\right)}{\sum_{k=1}^{K}\;\sum_{g=1}^{G}\;\psi_{ngk,1}^{\left(t\right)}\sum_{d=1}^{D_{ng}}\;y_{ngd}/D_{n}+\sum_{k=1}^{K}\;\sum_{g=1}^{G}\;\psi_{ngk,0}^{\left(t\right)}\left(1\;-\;\sum_{d=1}^{D_{ng}}\;y_{ngd}/D_{n}\right)}\\ \;=\frac{\sum_{g=1}^{G}\;\psi_{ngk,1}^{\left(t\right)}\beta_{ng}+\sum_{g=1}^{G}\;\psi_{ngk,0}^{\left(t\right)}\left(1\;-\;\beta_{ng}\right)}{\sum_{k=1}^{K}\;\sum_{g=1}^{G}\;\psi_{ngk,1}^{\left(t\right)}\beta_{ng}+\sum_{k=1}^{K}\;\sum_{g=1}^{G}\;\psi_{ngk,0}^{\left(t\right)}\left(1\;-\;\beta_{ng}\right)}, \end{array}$$

where $\beta_{ng}$ is the observed beta value in array bulk data. Thus we can extend the estimation of $\theta_{nk}$ when the bulk data is in the form of array bulk data after the rescaling. We found that applying quantile normalization to both the reference and bulk DNAm data helped stabilize the results. Therefore, we used joint quantile normalization as part of our data preprocessing.

### 2.3 Multi-Omics Deconvolution

In practice, we have observed variations in the results of deconvolution from different types of omics data. These challenges motivated us to investigate the potential of using information from other omics data sources to improve the accuracy of cellular fraction estimates from a single omics data source.

**Figure 2:**
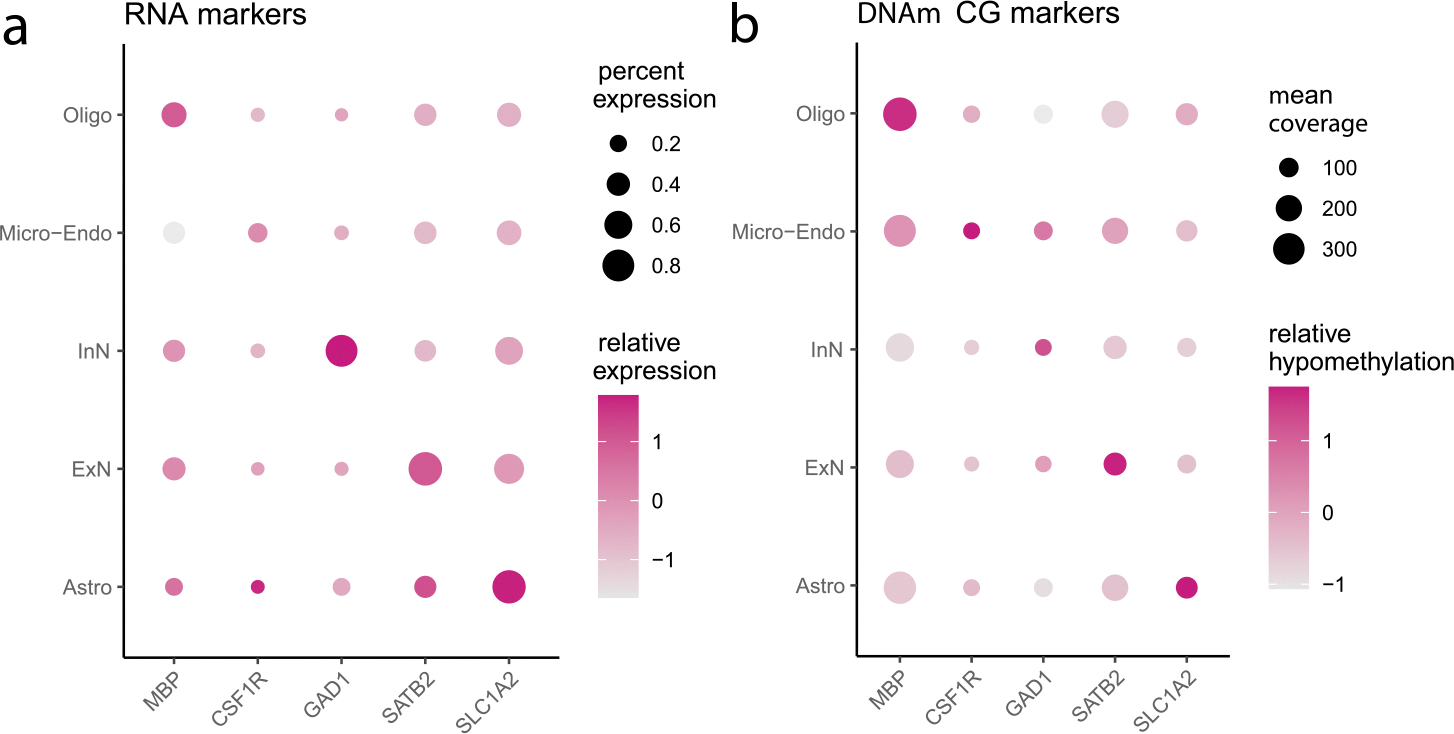
Canonical markers in single-cell RNA-seq (**a**) and scDNAm (**b**) using single-cell omics data from Luo et al. (2022). The five markers on the x-axis correspond to the five cell types on the y-axis, respectively.

Multi-omics data have several characteristics that make them useful for this purpose: 1) the multi-omics data from a tissue sample share the same *true* cell composition; 2) the *estimated* cell type fractions across observed multi-omics data from the same tissue region of an individual are similar, while affected by sampling and technical variability across omics data; 3) strong markers are shared across omics data (Teschendorff et al., 2020). Using single-cell multi-omics data, we observed similar cell-type marker patterns across different data types, such as gene expression and DNAm (Figure 2). These results justify the multi-omics deconvolution from multi-omics references, given that some markers may appear weak in specific data types because of technical variability.

Based on the results from real data, estimates relying solely on DNAm or RNA-seq often diverge significantly from the true cell fractions, though in different directions. This highlights the need for a multimodal approach that integrates both RNA-seq and DNAm data to achieve more accurate results. While a unified LDA model may provide a robust framework for combining these two modalities, our real data analysis revealed that a simple average of the DNAm-derived and RNA-seq-derived fractions produces the more reliable estimates. This may be due to the fact that we are inferring from similar models and the estimated cell fractions have comparable scales. The average serves as a sufficiently effective method to achieve robust and consistent cellular fraction estimates.

## 3 Results

### 3.1 Validating EMixed-DNAm Using Sorted-Cell Data

In this section, we describe the use of the EMixed method for cellular deconvolution in terms of DNAm data. To assess EMixed’s capability in distinguishing and quantifying major brain cell types, we embarked on a comprehensive evaluation, leveraging sorted datasets from seminal studies. We incorporated datasets from Guintivano et al. (2013) and Gasparoni et al. (2018), which include DNAm samples from sorted NeuN+ neurons and non-neuronal (NeuN−) cells. These curated datasets, with their definitive cell-type fractions, serve as an ideal benchmark for precisely evaluating EMixed’s performance.

In our comparative study, EMixed was analyzed alongside scMD (Cai et al., 2024), EpiSCORE (Teschendorff et al., 2020), and HiBED (Zhang et al., 2023), focusing on its ability to accurately deconvolve cell types across various datasets. scMD leverages information from scDNAm data to construct scDNAm signatures and perform deconvolution using the core functionality of EnsDeconv (Cai et al., 2022). EpiSCORE utilizes a reference derived from single-cell RNA sequencing (scRNA-seq) to impute DNA methylation at promoter regions of marker genes, followed by deconvolution based on these imputed signatures. HiBED employs a hierarchical modeling approach to deconvolve brain tissues into their major brain cell types with sorted-cell references. For EMixed, we used a reference signature generated from scMD, which provides a strong foundation for DNAm-based deconvolution.

**Figure 3:**
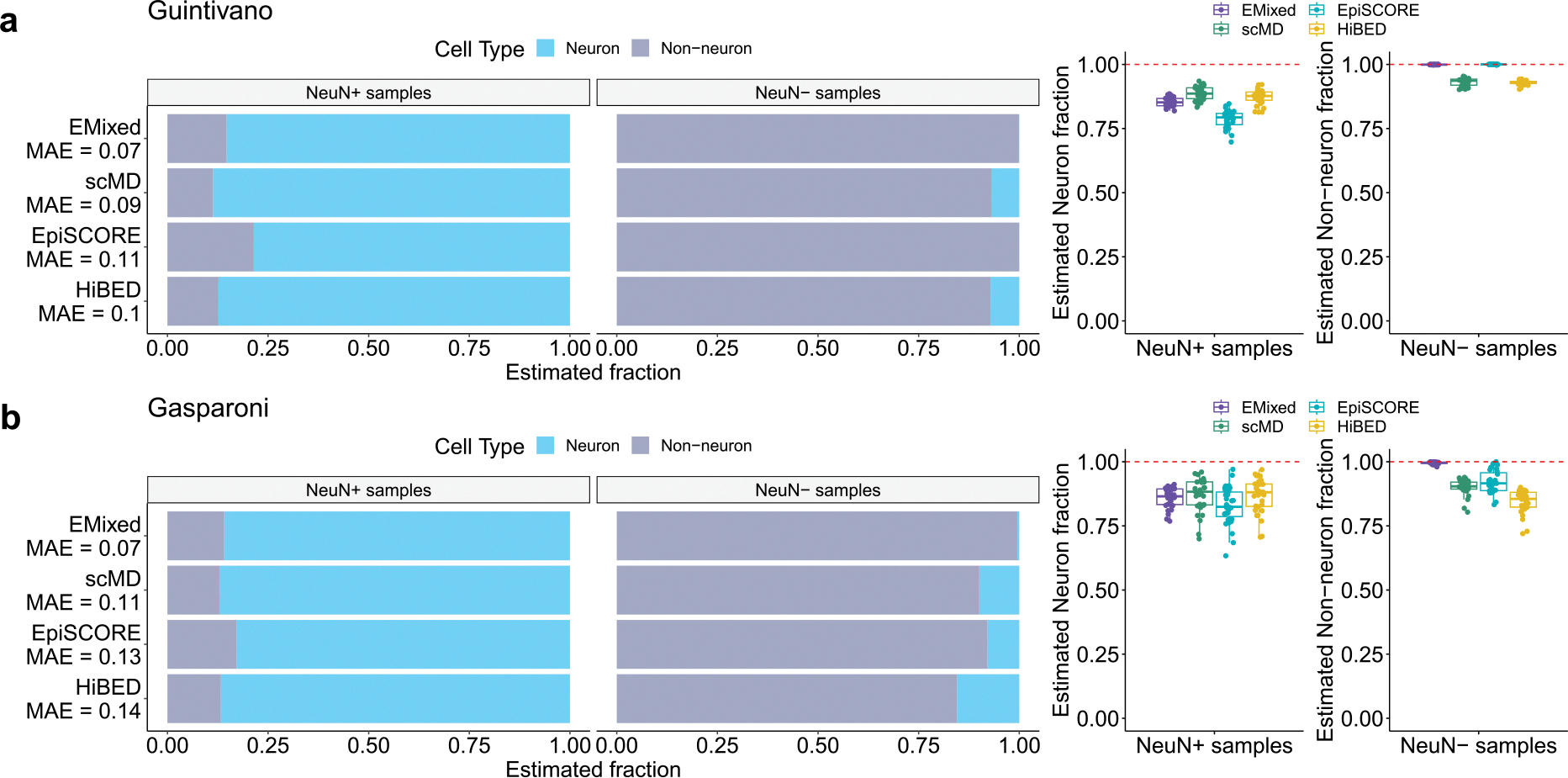
Validating cell-type DNAm signature from scDNAm data on sorted-cell data. **a**, validation on Guintivano et al. (2013). Bar plots show the mean estimated cellular fractions across NeuN+ and NeuN− samples. A comparison of EMixed, scMD, EpiSCORE, and HiBED is presented. **b**, validation on Gasparoni et al. (2018). Box plots show the cellular fractions in sorted NeuN+ and NeuN− samples. For benchmarking, the fraction estimates of cell subtypes were aggregated to generate the fractions of broader cell types.

The evaluations on the 450k array-based samples from Guintivano et al. (2013) and Gasparoni et al. (2018), illustrated in Figures 3a and 3b respectively, highlighted EMixed’s precision in deconvolving both NeuN+ and NeuN− samples. This underlines EMixed’s adaptability and superior performance in brain cell-type deconvolution across different methods. In comparison to other methods, EMixed not only nearly perfectly estimates all NeuN− samples but also achieves the lowest mean absolute error (MAE), positioning it as a highly efficient and accurate tool for cell-type deconvolution in neuroscience research.

### 3.2 Consistent Cellular Fractions from DNAm and RNA-Seq

We applied EMixed to a bulk blood dataset from the Epigenetic Variation and Childhood Asthma in Puerto Ricans (EVA-PR) (Chen et al., 2017; Jiang et al., 2019). The EVA-PR dataset of 220 samples provides measured cell type fractions, which can serve as ground truth, along with matched measurements of two omics data types: DNAm and gene expression. Specifically, this study offers paired quantified bulk data for both DNAm and RNA-seq, enabling direct comparison.

The core hypothesis of our analysis is that a high concordance between cellular fractions estimated from DNAm and RNA-seq data should be observed for the same tissue samples, given that both are derived from a common cellular composition. To assess this, we applied EMixed separately to RNA-seq and DNAm data. We conducted a comparative deconvolution of the EVAPR bulk DNAm and RNA-seq data, ensuring that all methods utilized the same reference signature for consistency. The performance of each method was evaluated using the concordance correlation coefficient (CCC) (Lawrence and Lin, 1989) between the RNA-seq and DNAm estimated fractions.

For DNAm, we utilized the Salas et al. (2022) reference and followed the pipeline provided in the minfi R package to construct DNAm signature matrices. For RNA-seq, we employed the lm22 dataset (Newman et al., 2015) as the reference. Both references are widely used in the field and are recognized for their robustness and reliability.

EMixed demonstrated superior performance, achieving a mean CCC of 0.52, indicating a higher level of agreement between RNA-seq and DNAm estimates compared with other methods. In contrast, CIBERSORT (Newman et al., 2015) produced a mean CCC of 0.14, with EPIC (Racle et al., 2017) and DCQ (Altboum et al., 2014) showing CCC values of 0.2 and 0.02, respectively. These findings suggest that EMixed provides a more robust and reliable concordance between RNA-seq and DNAm estimates, likely due to its LDA model-based approach, which yields more consistent results across modalities.

Notably, for rare cell types such as eosinophils, EMixed also achieved a stronger concordance, with a Spearman’s correlation of 0.63, while CIBERSORT, EPIC, and DCQ exhibited lower correlations of 0.41, 0.24, and 0.025, respectively. This highlights EMixed’s ability to more accurately capture cellular composition across both abundant and rare cell types, further establishing its reliability in multi-omics deconvolution.

### 3.3 Improved Results Using Multi-Omics Data and Measured Cell Fractions

In this section, we summarize the performance of the EMixed method across both single- and multi-modality deconvolution tasks. We applied EMixed to the EVA-PR dataset, which contains measured cell type fractions as ground truth, along with matched bulk DNAm and gene expression data. Our evaluation addressed two key components: first, the performance of EMixed-DNAm and EMixed-RNA, where the method was applied separately to DNAm and RNA-seq data, and second, the performance of EMixed, which integrates both data types. To quantify accuracy, we computed the mean CCC for each cell type and compared the estimated fractions to the measured ground truth. This allowed us to evaluate how well EMixed performed in both single-modality and multi-modality settings.

**Figure 5:**
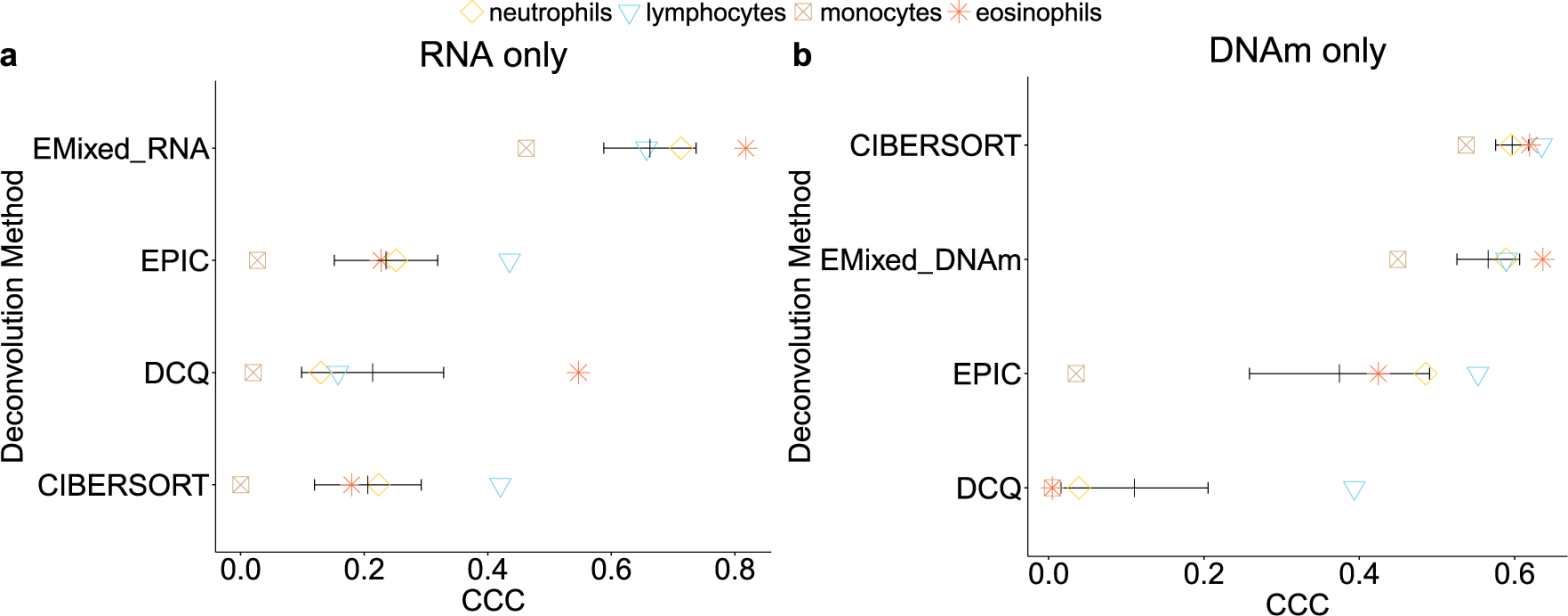
Benchmarking of EMixed and single-omics deconvolution methods for single modality: RNA (a) and DNAm (b) only. We compared different deconvolution methods and EMixed on the EVAPR data. For each method, each dot denotes one CCC for each cell type. The black vertical line shows the mean CCC, and the horizontal lines present the mean ± standard error of the mean. EMixed_DNAm and EMixed_RNA are the results of applying EMixed using DNAm and RNA data only, respectively.

**Figure 6:**
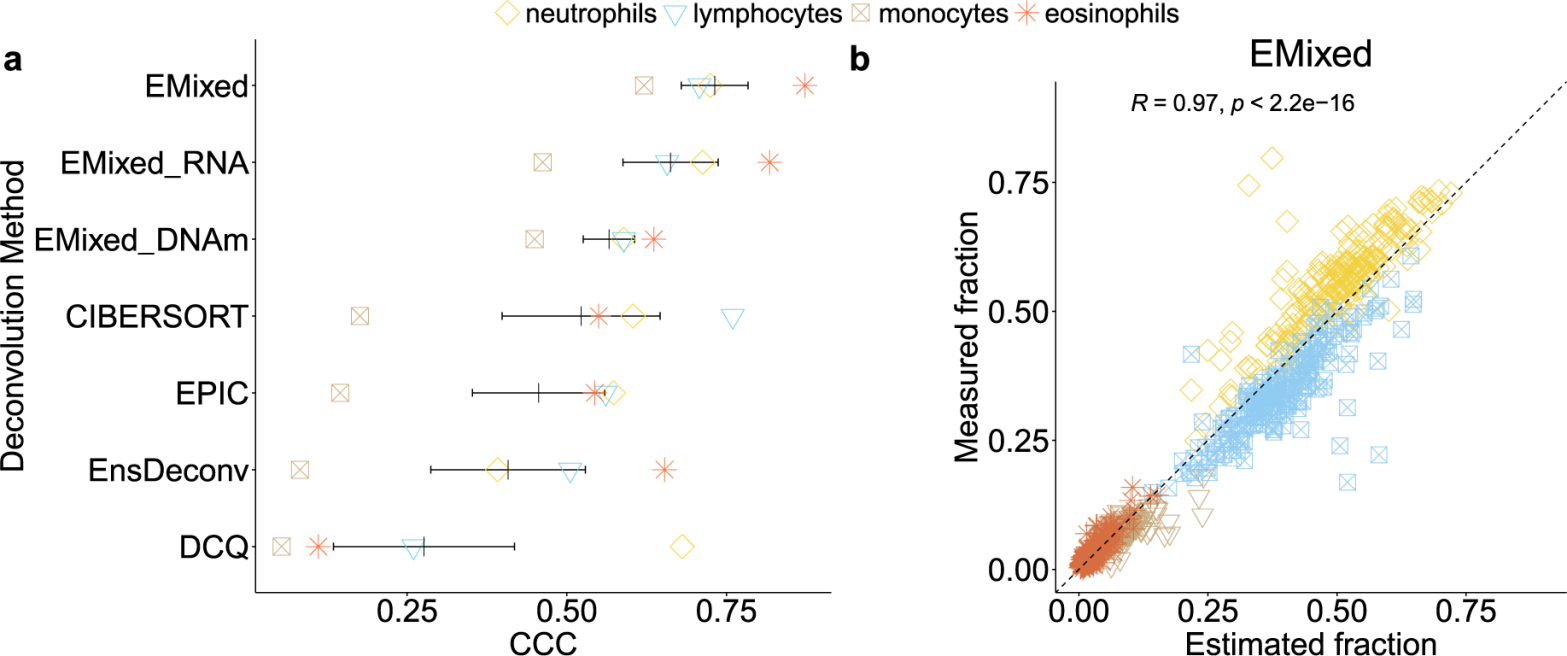
Benchmarking of EMixed and single-omics deconvolution methods on the EVAPR data. **a**. CCC for each method. Each dot denotes one CCC for each cell type. The black vertical line shows the mean CCC, and the horizontal lines present the mean ± standard error of the mean. EMixed_DNAm and EMixed_RNA are the results of applying EMixed using DNAm and RNA data only, respectively. For single-omics methods, we estimate the cell fractions from each omics and average the fractions from DNAm and RNA. **b**. Scatterplots of measured and EMixed estimated cell fractions.

As illustrated in Figure 5, EMixed achieved high concordance in single-modality deconvolution, with DNAm and RNA-seq each yielding strong mean CCC values across cell types. EMixed-DNAm achieved a mean CCC close to 0.6, while EMixed-RNA exceeded 0.7, demonstrating that the method is effective when using either modality independently. However, when combining both modalities in the EMixed model, the results further improved, as shown in Figure 6. EMixed achieved the highest concordance, with mean CCC values approaching 0.73 across all cell types, significantly outperforming other methods such as CIBERSORT, EPIC, and DCQ.

Additionally, EMixed demonstrated particularly strong performance for rarer cell types, such as eosinophils. The multi-modality approach achieved a CCC of 0.87, significantly outperforming the DNAm-only method, which yielded a CCC of 0.64. In contrast, other methods like CIBERSORT, EPIC, and DCQ exhibited much lower concordance for eosinophils. A similar trend was observed for neutrophils, where the single-modality approaches yielded a CCC of 0.59 for DNAm and 0.71 for RNA-seq. However, when the two modalities were combined, the CCC increased to 0.72. For cell types like monocytes, which showed lower concordance in single-omics methods (CCC of 0.45 for DNAm and 0.46 for RNA-seq), the multi-omics approach raised the CCC to 0.62. These results emphasize the advantages of integrating RNA-seq and DNAm data to improve cellular fraction estimates, particularly for cell types with weaker signals in single-modality approaches.

These results clearly demonstrate that while EMixed performs robustly in single-modality deconvolution, its ability to integrate RNA-seq and DNAm data in the multi-modality setting provides an added advantage, yielding more reliable and accurate cellular fraction estimates. This establishes EMixed as a highly versatile and powerful tool for multi-omics deconvolution, capable of capitalizing on complementary information across different omics layers to improve deconvolution accuracy.

### 3.4 EMixed Delivers Biologically Meaningful Results for Differential Cell Fraction Analysis

**Figure 7:**
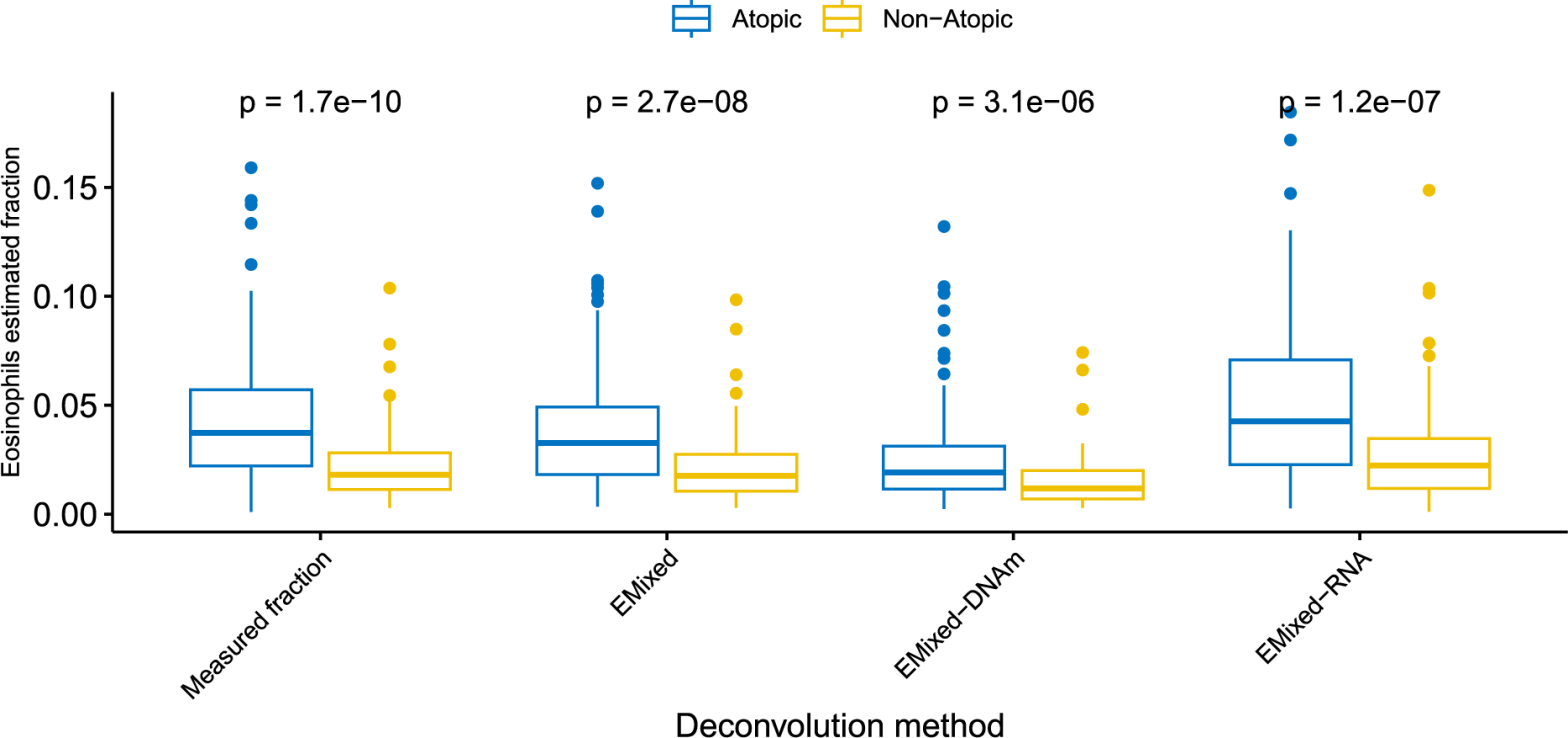
Analysis of differential cell type fraction with EVAPR data. Comparison using different deconvolution results for atopic and non-atopic samples.

To illustrate the application of our method in downstream analyses, we employed the EVA-PR dataset, which includes a biomarker of atopy (⩾ 1 positive IgE to common allergens). A key scientific question is whether there are differences in cell type fractions between atopic and non-atopic individuals. Our analysis of the measured cell type fractions revealed a significant difference in eosinophils, a type of white blood cells typically elevated in atopic individuals (two-sided Wilcoxon test, p-value = 1.7 × 10^−10^). Using EMixed to estimate cell type proportions, we replicated this significant finding for eosinophils across both multi-modality and single-modality analyses, with no significant differences observed in other cell types (Figure 7).

## 4 Discussion

In summary, we introduce EMixed, a novel deconvolution method designed to leverage multi-omics data—specifically RNA expression (bulk RNA-seq) and DNA methylation (bulk DNAm)—to more accurately estimate cellular compositions in heterogeneous biological samples. Traditional deconvolution methods typically rely on single-omics data, which limits their ability to capture the full complexity of biological samples due to technical variability and incomplete information provided by any one modality. To address this limitation, EMixed employs LDA modeling for both RNA and DNAm data, and integrates these complementary modalities using an EM algorithm to generate more robust estimates of cellular fractions. While we explored a data-driven approach to determine the relative weights of DNA and RNA datasets, this approach did not yield improvements over assigning equal weights. As part of our future work, we plan to develop and refine methods for determining these weights more effectively, thereby optimizing multi-omics integration and further improving the accuracy of the estimates.

We validated EMixed’s performance using sorted-cell DNAm datasets from different studies, such as those by Guintivano and Gasparoni, which include NeuN+ (neuronal) and NeuN− (non-neuronal) cell data. EMixed outperformed other deconvolution methods like scMD, EpiSCORE, and HiBED, achieving the lowest MAE and demonstrating superior accuracy in deconvolving brain cell types.

EMixed’s performance was also benchmarked using the EVAPR blood dataset, which contains both DNAm and RNA-seq data. EMixed achieved high CCC in multi-omics deconvolution, outperforming other methods in estimating cellular fractions from both abundant and rare cell types, such as eosinophils. Additionally, downstream analysis using the EVAPR dataset revealed significant differences in eosinophil levels between atopic and non-atopic individuals, further validating EMixed’s ability to generate biologically meaningful results. Overall, EMixed represents a powerful tool for multi-omics deconvolution, offering improved accuracy and insights for both research and clinical applications.

One advantage of the LDA-based framework in EMixed is its potential to dynamically update reference signatures with those estimated from bulk data, enhancing its utility when predefined references are incomplete. However, initial attempts at implementing this have shown less reliable results, likely due to limited data. While the framework offers flexibility, the accuracy of updates depends on data quality and quantity. Future work could address this by incorporating more omics data, improving reference selection, and developing techniques for reliable updates, ultimately improving the robustness and accuracy of cellular fraction estimates.

In conclusion, EMixed offers a versatile and highly effective tool for cellular deconvolution by integrating multi-omics data. Its ability to capitalize on the complementary strengths of RNA and DNAm data makes it a valuable resource for improving the accuracy of cellular composition estimates in complex biological samples, providing deeper insights for both research and clinical applications. This work establishes EMixed as a significant advancement in the field of multi-omics deconvolution, with the potential to enhance our understanding of tissue biology across various contexts.

## Supplementary Material

R package EMixed is publicly hosted on GitHub (https://github.com/manqicai/EMixed)

## Figures and Tables

**Figure 4:**
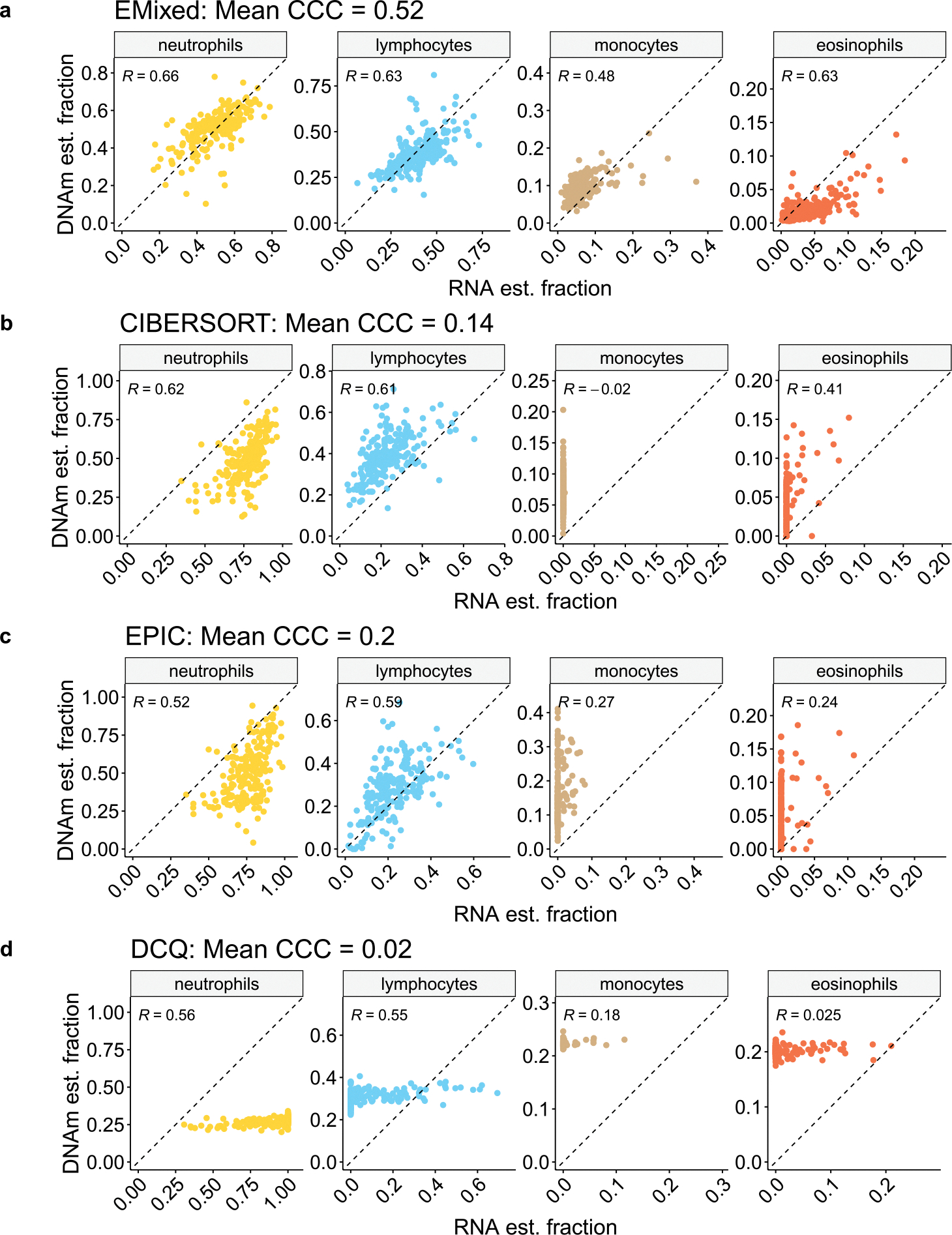
Comparison of cell type estimates from DNAm and RNA data using EMixed (a), CIBERSORT (b), EPIC (C), and DCQ (d). Scatter plots showcase the relationship between the estimated cell fractions from RNA data (x-axis) and DNAm data (y-axis) of bulk EVAPR data.
